# Inhibition of Small-Conductance, Ca^2+^-Activated K^+^ Current by Ondansetron

**DOI:** 10.3389/fphar.2021.651267

**Published:** 2021-04-22

**Authors:** Shuai Guo, Zhenhui Chen, Peng-Sheng Chen, Michael Rubart

**Affiliations:** ^1^ Division of Cardiology, Department of Medicine, The Krannert Institute of Cardiology, Indiana University School of Medicine, Indianapolis, IN, United States; ^2^ Department of Cardiology, Smidt Heart Institute, Cedars-Sinai Medical Center, Los Angeles, CA, United States; ^3^ Wells Center for Pediatric Research, Department of Pediatrics, Indiana University School of Medicine, Indianapolis, IN, United States

**Keywords:** ondansetron, small-conductance Ca^2+^ -activated K^+^ channel, voltage-clamp technique, apamin, HEK 293 cells, transfection

## Abstract

**Background:** Small-conductance Ca^2+^-activated K^+^ channels (SK channels) have been proposed as antiarrhythmic targets for the treatment of atrial fibrillation. We previously demonstrated that the 5-HT_3_ receptor antagonist ondansetron inhibits heterologously expressed, human SK2 (hSK2) currents as well as native cardiac SK currents in a physiological extra-/intracellular [K^+^] gradient at therapeutic (i.e., sub-micromolar) concentrations. A recent study, using symmetrical [K^+^] conditions, challenged this result. The goal of the present study was to revisit the inhibitory effect of ondansetron on hSK2-mediated currents in symmetrical [K^+^] conditions.

**Experimental Approach:** The whole-cell patch clamp technique was used to investigate the effects of ondansetron and apamin on hSK2-mediated currents expressed in HEK 293 cells. Currents were measured in symmetrical [K^+^] conditions in the presence of 100 nM [Ca^2+^]_o_.

**Results:** Expression of hSK2 produced inwardly rectifying whole-cell currents in the presence of 400 nM free cytosolic Ca^2+^. Ondansetron inhibited whole-cell hSK2 currents with *IC*_*50*_ values of 154 and 113 nM at −80 and 40 mV, respectively. Macroscopic current inhibited by ondansetron and current inhibited by apamin exhibited inwardly rectifying current-voltage relationships with similar reversal potentials (apamin, ∼5 mV and ondansetron, ∼2 mV). Ondansetron (1 μM) in the continuing presence of apamin (100 nM) had no effect on hSK2-mediated whole-cell currents. Wild-type HEK 293 cells did not express ondansetron- or apamin-sensitive currents.

**Conclusion:** Ondansetron in sub-micromolar concentrations inhibits hSK2 currents even under altered ionic conditions.

## Introduction

Small-conductance Ca^2+^-activated K^+^ channels (SK channels) have been proposed as antiarrhythmic targets for the treatment of atrial fibrillation (Heijman and Dobrev, 2017; Ravens and Odening, 2017). Three SK channel isoforms (SK1, SK2 and SK3, which are encoded by the *KCNN1*, *KCNN2* and *KCNN3* genes, respectively) are expressed in the mammalian heart (Tuteja et al., 2005). Under physiological conditions, functional SK channels are predominately present in the atria and significantly contribute to atrial refractoriness and excitability (Xu et al., 2003; Li et al., 2009). In addition to the bee-venom toxin apamin, which selectively blocks SK channels at pico-to nanomolar concentrations (Stocker, 2004; Yu et al., 2014), a number of pharmacological SK channel inhibitors have been developed and shown to reduce susceptibility to atrial fibrillation in animal models (Skibsbye et al., 2011; Skibsbye et al., 2014; El-Haou et al., 2015; Diness et al., 2017; Ravens and Odening, 2017). We previously demonstrated that the 5-HT_3_ receptor antagonist ondansetron, a widely prescribed antiemetic, blocks heterologously expressed, human SK2 (hSK2) channels at therapeutic (i.e., sub-micromolar) concentrations (Ko et al., 2018), suggesting its possible repurpose as an atrial-selective antiarrhythmic drug. A recent study by Kirchhoff and co-workers challenged our findings reporting that ondansetron at micromolar concentrations had no significant effect on hSK2-mediated currents expressed in human embryonic kidney (HEK) 293 cells (Kirchhoff et al., 2019). Since ionic conditions for hSK2 current measurements that Kirchhoff et al. used (symmetrical [K^+^], presence of extracellular Ca^2+^) were different from those used in our previous experiments (physiological extra-/intracellular [K^+^] gradient, absence of extracellular Ca^2+^), the goal of the present study was to re-evaluate the efficacy of ondansetron in inhibiting hSK2-mediated currents expressed in HEK 293 cells under ionic conditions replicating those employed by Kirchhoff et al. Our results indicate that ondansetron retains its inhibitory effect on hSK2 channels even under altered ionic conditions.

## Materials and Methods

### Human Embryonic Kidney 293 Cells and Plasmid Transfection

Human embryonic kidney (HEK) 293 cells (American Type Culture Collection, Manassas, VA, United States) were cultured in Iscove’s modified Dulbecco’s medium (ThermoFisher Scientific, Waltham, MA, United States) supplemented with 10% FBS and 1% penicillin-streptomycin in a 95% O_2_-5% CO_2_ incubator at 37°C. HEK 293 cells were transfected with 1 μg of a plasmid encoding human SK2 (Gene Bank Accession #NM_021614.2; OriGene, Rockville, MD, United States) (Turker et al., 2013) and internal ribosome entry site-enhanced green fluorescent protein (pSK2-IRES-eGFP) using Effectene Transfection Reagent (Qiagen). Cells expressing SK2 were identified by virtue of their green fluorescence and used for patch-clamp experiments.

### Electrophysiology

Whole-cell Ca^2+^-activated K^+^ current was recorded from transfected HEK 293 cells at room temperature using the patch-clamp technique in the ruptured patch configuration. The extracellular solution contained (in mmol/L) 0.1 CaCl_2_, 3 MgCl_2_, 154 KCl, 10 HEPES, and 10 glucose (pH = 7.4 and 285–295 mOsm). The internal solution contained (in mmol/L) 8.106 CaCl_2_ (yielding a free Ca2+ concentration of 400 nM, using the Maxchelator software by C. Patton of Stanford University as we have previously described (Ko, 2018)), 1.167 MgCl_2_, 10 EGTA, 108 KCl, 10 HEPES, 31.25/10 KOH/EGTA, and 15 KOH (pH = 7.2). Pipette resistances ranged from 3 to 5 MΩ when filled with the pipette solution. After a gigaohm seal had been achieved, the test pulse current was nulled by adjusting the pipette capacitance compensation. After break in, the whole cell charging transient was nulled by adjusting whole cell capacitance and series resistance. Currents were elicited using a voltage ramp from −80 to 80 mV (0.8 mV/ms) from a holding potential of 0 mV. Voltage ramps were repeated every 2 s. To isolate apamin- or ondansetron-sensitive currents, extracellular solution containing apamin (100 nM) or ondansetron (0.1, 0.5, 1.0 or 5.0 μM) was applied during continuous recordings, and the difference currents between the baseline and the apamin-/ondansetron-containing solution were calculated to be the apamin- or ondansetron-sensitive currents (*I*_*apamin*_ and *I*_*ondan*_, respectively). To determine the concentration-inhibition relationship for ondansetron of expressed hSK2 currents, cells were first exposed to a given concentration of ondansetron followed by exposure to 100 nM apamin in the continual presence of ondansetron. The amplitude of *I*_*ondan*_ was expressed as *I*_*ondan*_/(*I*_*ondan*_ + *I*_*apamin*_) and fit to an equation of the form

$$1-\frac{I_{ondan}}{I_{ondan}+I_{apamin}}=1-\frac{I_{\;ondan,\max}}{I_{ondan,\max}+I_{apamin}}+\;\frac{1-\;\left(1-\left(I_{ondan,\max}/\left(I_{ondan,\max}+I_{apamin}\right)\right)\right)\;}{1+\;10^{\left(\log IC_{50\;}-X\right)}}$$

where *X* is the concentration of ondansetron (expressed in logarithmic units), *I*_*ondan,max*_ is the maximal *I*_*ondan*_ obtainable, and *IC*_50_ is the concentration of ondansetron at which 1-*I*_*ondan*_/(*I*_*ondan*_ + *I*_*apamin*_) equals 0.5.

Currents were normalized to cell capacitance to obtain current density. Series resistance was electronically compensated by 70–80%. Voltage-clamp commands were generated with an Axopatch 200B amplifier/Digidata 1440A acquisition system using pCLAMP10 software (Molecular Devices). The electrical signal was filtered with a built-in four-pole low-pass Bessel filter at 1 kHz and digitized at 5 kHz. Whole cell recordings were analyzed using Clampfit 10.2 software (Molecular Devices) and Sigmaplot software (Systat Software, San Jose, CA, United States).

### Drugs

Apamin and ondansetron were purchased from Tocris Bioscience. All other chemicals were purchased from Sigma-Aldrich (St. Louis, MO, United States). Apamin and ondansetron were dissolved in double-distilled H_2_O to make stock solutions at concentrations of 10 mM/L and 1 mM/L, respectively. These stock solutions were stored at −20°C and aliquots were solubilized at the desired concentration on the day of the experiment.

### Statistics

All numerical values are expressed as mean ± S.E.M. Statistical analysis was performed using Sigmaplot software. Data were analyzed with a *t*-test where appropriate.

## Results

### Wild Type Human Embryonic Kidney 293 Cells Do Not Express Apamin- or Ondansetron-Sensitive Currents

**FIGURE 1:**
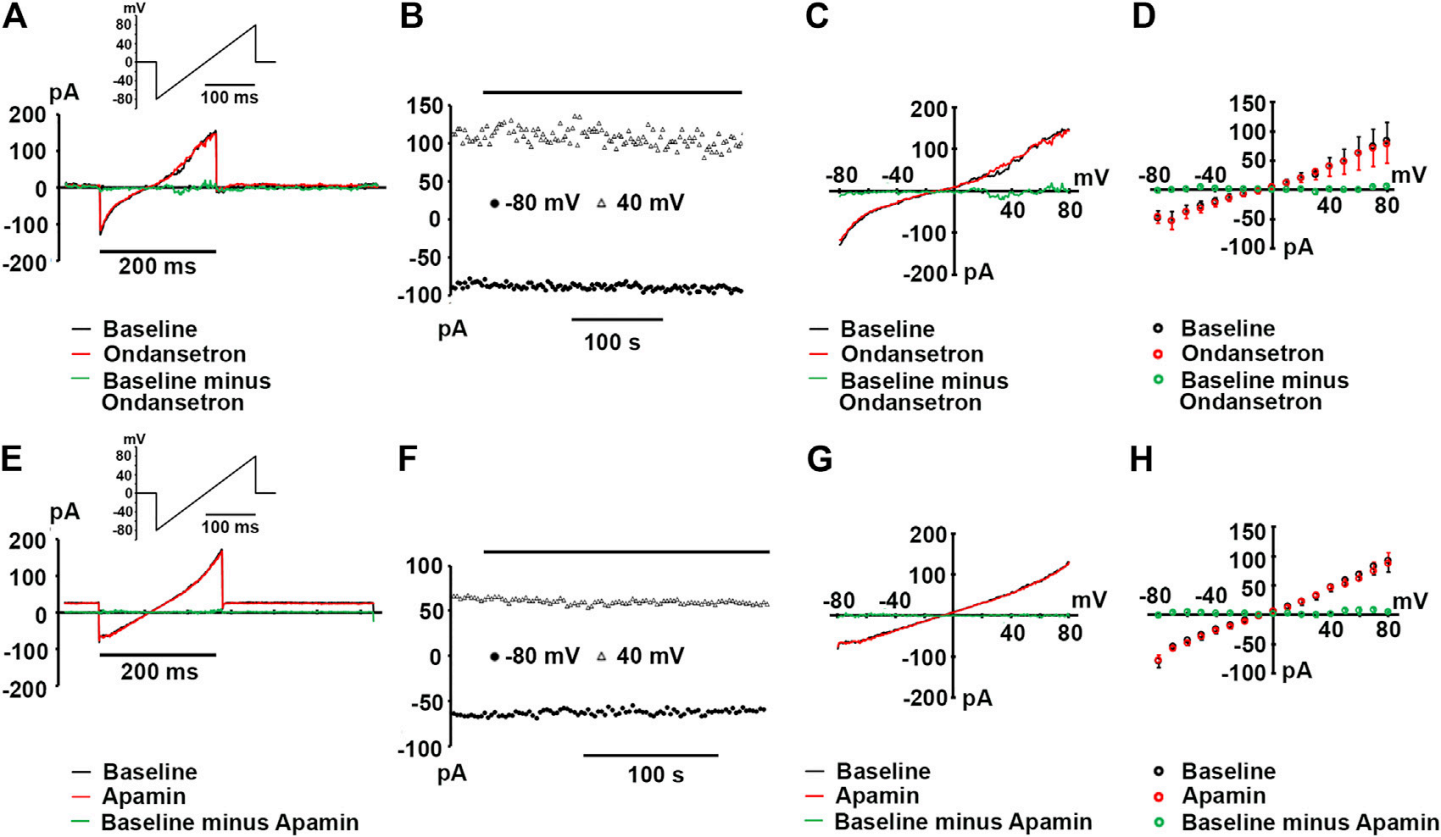
Wild type HEK 293 cells lack apamin- or ondansetron-sensitive currents. **(A,E)** Representative whole-cell currents in response to a voltage ramp from -80 to 80 mV recorded at baseline and following >3-min exposures to ondansetron [(1 μM); **(A)**] or apamin [(100 nM); **(E)**]; insets: voltage-clamp protocols. **(B,F)** Plots of inward current amplitude at −80 and 40 mV as a function of time at baseline and during ondansetron or apamin exposure for the cells in A and E, respectively. Times of drug applications are indicated by the horizontal lines. **(C,G)** *I-V* relationships for the baseline current, current in the presence of ondansetron **(C)** or apamin **(G)**, and the ondansetron- or apamin-sensitive currents for the cells in **(A,E)**, respectively. The ondansetron- and apamin-sensitive currents were obtained by digitally subtracting the currents recorded in the presence of either drug from those recorded in their absence. **(D,H)** Summary of *I-V* relationships at baseline and during ondansetron **(D)** or apamin **(H)** treatment. Values represent mean ± SEM for 3 cells per treatment. Pipette solutions for all experiments shown in **(A)** through F contained 0.4 μM free Ca^2+^.

In order to rule out the possibility that our results might be confounded by the endogenous expression of apamin- or ondansetron-sensitive currents in HEK 293 cells, we made patch-clamp recordings from wild type HEK 293 cells. Figures 1A,E show representative ramp currents recorded at baseline (before application of apamin or ondansetron, black traces) and after apamin or ondansetron application (red traces). No significant alterations in whole-cell currents by apamin or ondansetron were observed. Plots of peak inward currents at −80 and 40 mV as a function of time (Figures 1B,F) as well as whole-cell *I-V* relationships (Figures 1C,G) for the cells shown in Figures 1A,E illustrate the absence of significant apamin or ondansetron effects on endogenous currents. Summary data for endogenous currents at test potentials ranging from −80 to +80 mV are shown in Figures 1D,H. Collectively, these data suggest that there is no detectable expression of apamin- or ondansetron-sensitive currents in wild type HEK 293 cells used in the present study.

### Block of hSK2-Mediated Currents by Apamin and Ondansetron

**FIGURE 2:**
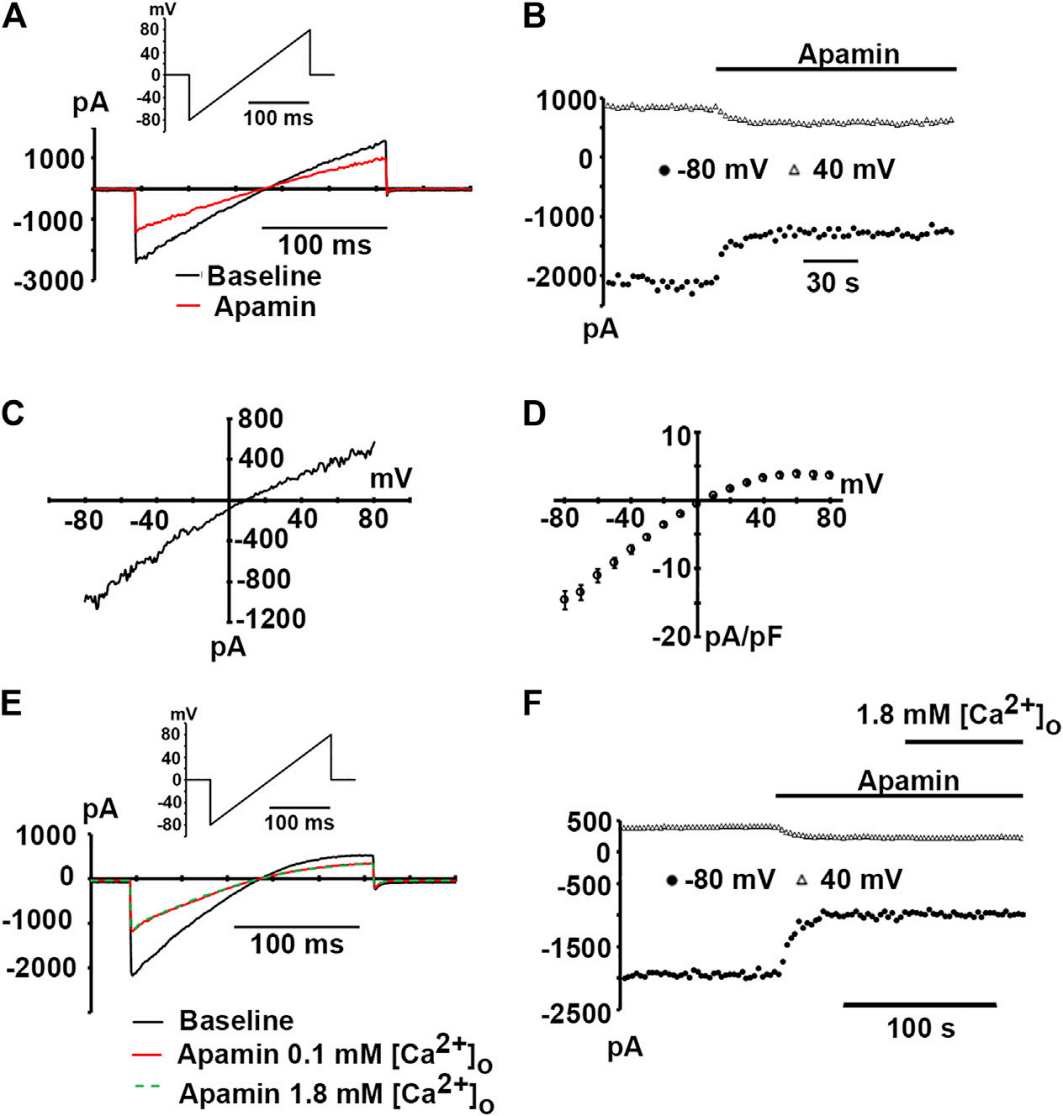
Apamin-sensitive, inwardly rectifying currents in hSK2-transfected HEK 293 cells. **(A)** Representative whole-cell currents in response to a voltage ramp from −80 to 80 mV (inset) at baseline and following a ∼2-min continuous exposure to apamin (100 nM). **(B)** Plot of inward current amplitude at −80 and 40 mV as a function of time for the cell in A at baseline and during apamin treatment. Apamin reduced current amplitude by ∼80%. **(C)** *I-V* relationship for the apamin-sensitive current for the cell in A. The ratio between current amplitude at 80 mV and that at -80 mV (*I*_80_/*I*_−80_) was 0.25, indicating strong inward rectification. **(D)** Average apamin-sensitive current densities plotted as a function of voltage with error bars representing SEM from 5 cells. **(E)** Representative whole-cell currents in response to voltage ramps from −80 to 80 mV (inset) at baseline and following exposure to apamin (100 nM) in 0.1 mM (red trace) and 1.8 mM [Ca^2+^]_o_ (dashed green trace). Traces are representative of two experiments. (**F)** Plot of inward current amplitude measured at −80 and 40 mV as a function of time for the cell in **(E)**.

Expression of hSK2 produced inwardly rectifying whole-cell currents in the presence of 400 nM free cytosolic Ca^2+^ (Figure 2A, black trace). hSK2-mediated currents were blocked by 100 nM apamin (Figure 2A, red trace, and Figure 2B), but block was incomplete despite addition of a saturating concentration of the bee venom toxin. On average, 100 nM apamin reduced currents measured at −80 and 40 mV by 47.3 ± 4.4% and 41.7 ± 4.8%, respectively (8 hSK2-HEK 293 cells). These values are in close agreement with those previously reported for heterologously expressed whole-cell rat SK2 currents measured in symmetrical [K^+^] conditions (Lamy et al., 2010), but significantly smaller than those observed for outside-out macropatch rat SK2 currents (Weatherall et al., 2011). Macroscopic apamin-sensitive currents exhibited inwardly rectifying current-voltage relationships with a reversal potential of 4.6 ± 1.2 mV (*n* = 5 cells; Figures 2C,D), which is close to the 0 mV Nernst potential for symmetrical [K^+^] conditions. Apamin block of expressed hSK2 currents was also assessed under physiological external [Ca^2+^] conditions in 2 hSK2-HEK 293 cells. Increasing external [Ca^2+^] from 0.1 to 1.8 mM in the continual presence of 100 nM apamin did not change the magnitude of current inhibition (Figures 2E,F), suggesting that the concentration of external Ca^2+^ did not significantly affect the potency of apamin block.

**FIGURE 3:**
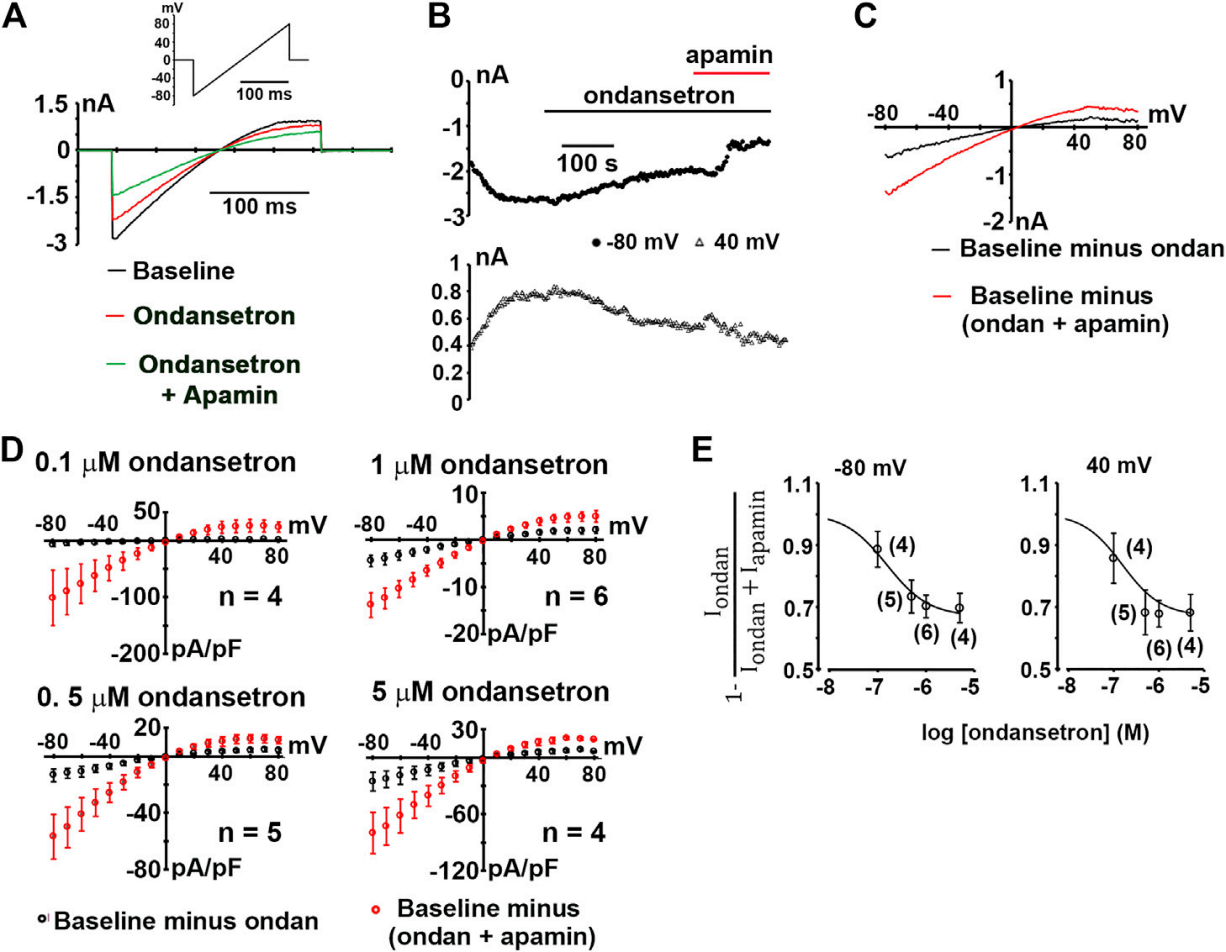
Inhibition of hSK2-mediated current by ondansetron. **(A)** Exemplary whole-cell currents in response to a voltage ramp from −80 to 80 mV (inset) at baseline and at the end of a ∼5-min exposure to ondansetron (1 μM). **(B)** Plot of inward current at −80 and 40 mV as a function of time for the cell in A at baseline, during ondansetron treatment and during combined exposure to ondansetron and apamin (100 nM). Horizontal lines demark application of ondansetron and apamin. **(C)**, *I-V* relationships for the baseline current, ondansetron-sensitive current, and the (ondansetron + apamin) - sensitive current for the cell in **(A)**. **(D)** Average densities of the ondansetron-sensitive and (ondansetron + apamin) - sensitive currents plotted as a function of voltage with error bars representing SEM. *n* indicates number of independent experiments. **(E)** Concentration-inhibition relationships for ondansetron inhibition of expressed hSK2 current. Numbers in parentheses indicate numbers of independent experiments per data point. Solid curves are best fits to a Hill equation.

**FIGURE 4:**
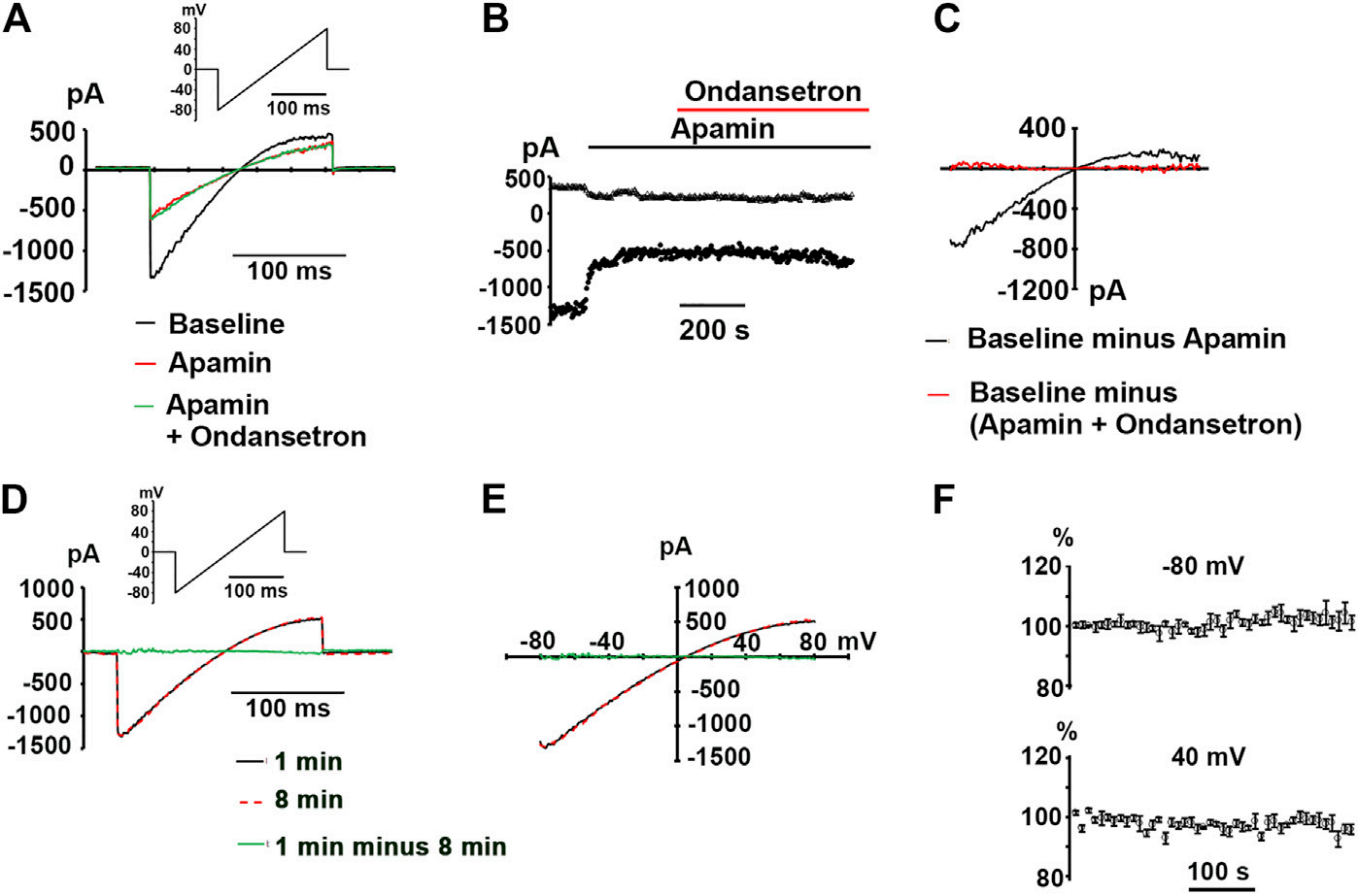
No alteration in whole-cell currents by ondansetron in apamin-pretreated hSK2-HEK 293 cells. **(A)** Representative ramp currents recorded at baseline, in response to apamin alone (100 nM) and in response to ondansetron (1 μM) in the continual presence of apamin. **(B)** Plot of inward current at −80 and 40 mV as a function of time for the same cell in A at baseline and during continuous exposure to apamin alone (100 nM) followed by exposure to ondansetron (1 μM) plus apamin (100 nM). Times of drug application are indicated by horizontal lines. **(C)** *I-V* relationships for the baseline current, ondansetron-sensitive current, and the (apamin + ondansetron) - sensitive current for the cell in **(A)**. **(D)** Whole-cell ramp current traces obtained from a voltage-clamped hSK2-HEK 293 cell. The interval between the two recordings was 8 min. **(E)** Superimposition of the *I-V* relationships for the 2 time points for the cell in **(D)**. **(F)** Time plots of current amplitude as a percentage of initial amplitude at −80 and 40 mV (mean ± SEM from 4 independent experiments). Currents were elicited by repetitive (0.2 Hz) 200-ms ramps from −80 to 80 mV over ∼8 min.

Next, we sought to examine whether ondansetron at a concentration of 1 μM inhibits hSK2-mediated currents using ionic conditions that are identical to those reported by Kirchhoff et al. Representative whole-cell current traces obtained from ramps from −80 to 80 mV imposed on a voltage-clamped HEK 293 cell expressing hSK2 are demonstrated in Figure 3A. Ondansetron produced a partial block of hSK2-mediated ramp current (red trace) and addition of apamin on top of ondansetron further reduced its magnitude (green trace). Typically, development of ondansetron block of hSK2-mediated ramp currents took considerably longer time (minutes) to reach steady-state compared to that of apamin-induced block (seconds), as exemplified in Figure 3B. The current-voltage relationships for current inhibited by ondansetron and for current inhibited by cumulatively applied apamin (Figures 3C,D) suggest that 1 μM ondansetron inhibited a current with properties of apamin-sensitive currents, i.e., a reversal potential (2.2 ± 2.4 mV, *n* = 6 cells.) close to *E*_*K*_ (0 mV) and an inwardly rectifying current-voltage relationship. The reversal potential of the ondansetron-sensitive current is not significantly different from that of the apamin-sensitive current (4.6 ± 1.2 mV, *n* = 5 cells; *p* = 0.4 by *t*-test). To determine the concentration-dependence of ondansetron inhibition, whole-cell ramp currents were measured at a single concentration of ondansetron (0.1, 0.5, 1 or 5 μM) followed by measurements in the presence of cumulatively applied apamin (100 nM). Figure 3D summarizes the *I-V* relationships for the ondansetron-sensitive component (*I*_*ondan*_) and the (ondansetron + apamin)-sensitive component (*I*_*apamin*_ + *I*_*ondan*_). Figure 3E shows the fractional block of SK2 currents, i.e., 1-[*I*_*ondan*_/(*I*_*ondan*_ + *I*_*apamin*_)], measured at −80 and 40 mV in response to increasing concentrations of the drug. Fitting the data to a Hill equation yielded *IC*_50_ values of 154 nM (−80 mV) and 113 nM (40 mV). Values for 1-[*I*_*ondan*_/(*I*_*ondan*_ + *I*_*apamin*_)] plateaued at around 0.7 for micromolar ondansetron concentrations, indicating that not more than ∼30% of the composite, i.e., (ondansetron + apamin)-sensitive, currents were blocked by saturating concentrations of ondansetron under the experimental conditions employed here. Exposure of two hSK2-transfected HEK 293 cells to 1 μM ondansetron in the continuing presence of 100 nM apamin had no effect on ramp currents (Figures 4A–C), suggesting that apamin occupies the ondansetron binding site or prevents it from binding. The latter finding further indicates that 1 μM ondansetron does not significantly affect native currents expressed in hSK2-HEK 293 cells, confirming our measurements in wild type HEK 293 cells (see Figure 1).

Finally, to exclude the possibility that our results are confounded by spontaneous “run-down” of macroscopic currents during extended recordings, ramp currents were elicited every 5 s for 8 min under control conditions in 4 voltage-clamped cells. Representative recordings shown in Figures 4D,E as well as plots of the whole-cell current amplitudes at −80 and 40 mV (normalized to their respective initial values) as a function of time (Figure 4F) illustrate that the magnitudes of repetitively induced ramp currents remained largely unchanged throughout ∼8-min monitoring periods. Taken together, our data suggest that sub-micromolar concentrations of ondansetron inhibit hSK2-mediated currents expressed in HEK 293 cells in ionic conditions that are identical to those used in the study by Kirchhoff et al.

## Discussion

We previously demonstrated, using physiological [K^+^] conditions, that ondansetron at sub-micromolar concentrations inhibits hSK2-mediated currents expressed in HEK 293 cells as well as native SK currents in mouse ventricular cardiomyocytes (Ko et al., 2018). The experiments presented here, utilizing symmetrical [K^+^], provides additional experimental evidence that ondansetron in sub-micromolar concentrations inhibits hSK2 currents heterologously expressed in HEK 293 cells. We found that ondansetron-sensitive whole-cell currents exhibit properties of apamin-sensitive whole-cell currents, i.e., an inwardly rectifying current-voltage relationship and a reversal potential close to the calculated *E*_*K*_.Ondansetron had no significant effect in the continuing presence of apamin, indicating that ondansetron targets hSK2 channels in the experimental conditions employed here. Our findings also exclude the possibility that ondansetron inhibits native currents expressed in wild type HEK 293 cells. These results are in agreement with our previous observations that ondansetron, when applied in the continuing presence of a saturating apamin concentration, had no effect on whole-cell currents of mouse ventricular myocytes (Ko et al., 2018), nor did it prolong ventricular repolarization of isolated perfused rabbit hearts exposed to low [K^+^]_o_ or isolated perfused failing rabbit hearts (Chan et al., 2015; Yin et al., 2020).

Our results contrast with those recently published by Kirchhoff and co-workers who used identical ionic conditions to determine the effect of ondansetron on hSK2 currents (Kirchhoff et al., 2019). These authors report that ondansetron failed to inhibit macroscopic currents in hSK2-transfected HEK 293 cells at concentrations up to 30 μM. The reasons underlying this apparent discrepancy are unclear. We previously demonstrated that the *p*. F503L SK2 variant heterologously expressed in HEK 293 cells exhibits reduced sensitivity to inhibition by ondansetron (Ko et al., 2018). Thus, it is possible that the nucleotide sequences of the hSK2 cDNAs that were used for HEK 293 cell transfection differed within segments encoding for the ondansetron-binding motif, altering the affinity of the hSK2 channel to ondansetron. Comparisons of the respective hSK2 cDNA sequences may be useful to obtain information about the SK2 channel motifs that confer ondansetron sensitivity. It is also possible that differences in the experimental protocol are responsible for the discrepant findings. Cells in the study by Kirchhoff et al. were first exposed to bicuculline, a blocker of Ca^2+^-activated K^+^ channels (Khawaled et al., 1999), followed by a brief wash out before ondansetron was applied. Incomplete washout of bicuculline before ondansetron exposure, i.e., recovery from bicuculline block overlapping with ondansetron inhibition, may have masked an inhibitory effect of ondansetron in the study by Kirchhoff et al. Finally, our experiments revealed slow kinetics of the development of ondansetron-induced SK current inhibition (see Figure 3) relative to the kinetics of apamin inhibition, suggesting the possibility that insufficient durations of ondansetron wash-in may have contributed to the reported lack of its effect on hSK2 currents.

Ondansetron at saturating concentrations suppressed maximally 30% of the (ondansetron + apamin)-sensitive current component under the experimental conditions employed here, indicating that ondansetron had lower inhibitory potency than apamin. This finding contrasts with our previous observation that sub-micromolar concentrations of ondansetron and apamin were equipotent in blocking native SK currents in mouse ventricular cardiomyocytes as well as in blocking hSK2 currents expressed in HEK 293 cells in a physiological [K^+^] gradient (Ko et al., 2018). We cannot readily explain this apparent discrepancy. Differences in ondansetron binding to and/or differences in ondansetron sensitivity of SK channels may be responsible, resulting from differences in ionic conditions that were used for SK current measurements. Importantly, however, we also demonstrated in the same study that ondansetron at a concentration of 100 nM effectively inhibits apamin-sensitive channels in the intact, isolated perfused mouse heart at physiological temperatures, supporting the drug’s utility for *in vivo* SK current inhibition.

*IC*_50_ values of 154 nM (at −80 mV) and 113 nM (at 40 mV) reported here correspond to ondansetron plasma concentrations of ∼45 and ∼33 ng/ml, respectively. Therapeutic dosages of oral or suppository ondansetron result in peak plasma concentrations ranging from ∼15 to ∼100 ng/ml (de Wit et al., 1996; Jann et al., 1998; Vandenberg et al., 2000), which would suffice to inhibit SK current. The sensitivity of SK channels to ondansetron in clinically relevant concentrations suggests its potential utility as an antiarrhythmic drug. Since under physiological conditions functional SK channels are predominantly present in the atria and control repolarization and excitability (Li et al., 2009; Skibsbye et al., 2014), ondansetron may selectively target atrial SK channels without evoking ventricular proarrhythmia. However, SK channels may importantly contribute to ventricular repolarization in the diseased heart (Chan et al., 2015; Hamilton et al., 2020; Yin et al., 2020), raising the possibility that ondansetron under pathological conditions increases the proarrhythmic risk.

## Conclusion

Our data indicate that ondansetron at sub-micromolar concentrations blocks hSK2-mediated currents expressed in HEK 293 cells even under altered ionic conditions, largely confirming our previous findings. They further support the notion that ondansetron can be repurposed as an antiarrhythmic agent for treatment of cardiac arrhythmias.

## Data Availability

The raw data supporting the conclusion of this article will be made available by the authors, without undue reservation.
